# Intact predictive motor sequence learning in autism spectrum disorder

**DOI:** 10.1038/s41598-021-00173-1

**Published:** 2021-10-19

**Authors:** A. J. Rybicki, J. M. Galea, B. A. Schuster, C. Hiles, C. Fabian, J. L. Cook

**Affiliations:** School of Psychology, University of Birmingham, Birmingham, B15 2TT UK

**Keywords:** Psychology, Human behaviour

## Abstract

Atypical motor learning has been suggested to underpin the development of motoric challenges (e.g., handwriting difficulties) in autism. Bayesian accounts of autistic cognition propose a mechanistic explanation for differences in the learning process in autism. Specifically, that autistic individuals overweight incoming, at the expense of prior, information and are thus less likely to (a) build stable expectations of upcoming events and (b) react to statistically surprising events. Although Bayesian accounts have been suggested to explain differences in learning across a range of domains, to date, such accounts have not been extended to motor learning. 28 autistic and 35 non-autistic controls (IQ > 70) completed a computerised task in which they learned sequences of actions. On occasional “surprising” trials, an expected action had to be replaced with an unexpected action. Sequence learning was indexed as the reaction time difference between blocks which featured a predictable sequence and those that did not. Surprise-related slowing was indexed as the reaction time difference between surprising and unsurprising trials. No differences in sequence-learning or surprise-related slowing were observed between the groups. Bayesian statistics provided anecdotal to moderate evidence to support the conclusion that sequence learning and surprise-related slowing were comparable between the two groups. We conclude that individuals with autism do not show atypicalities in response to surprising events in the context of motor sequence-learning. These data demand careful consideration of the way in which Bayesian accounts of autism can (and cannot) be extended to the domain of motor learning.

## Introduction

Autism spectrum disorder (ASD) is a neurodevelopmental disorder, characterized by restricted and repetitive interests and difficulties with social communication and interaction (American Psychiatric Association, 2013). While not considered a core diagnostic feature, over recent years the study of autistic body movements has gained traction^1–3^ and differences in the way autistic and non-autistic people move have been documented^4^. A number of studies have suggested that movement challenges in autism could stem from atypicalities in the motor learning process^5,6^. Evidence to support this comes from serial reaction time tasks, wherein participants execute a sequence of discrete movements over repeated trials, with motor sequence learning indexed as a reduction in response time for learned sequences^7,8^. Several studies report atypical sequence learning in autism^9,10^. Thus, a small but growing literature suggests that differences in autistic body movements may lie, not in the execution of learned movements, but in the learning process itself.

The claim that motor learning is different in autism resonates well with a broader literature arguing for general learning atypicalities. Current prominent accounts of autism^11,12^ propose that major characteristics can be explained by differences in Bayesian inference. Under Bayesian^13^, specifically predictive coding frameworks^14,15^, perception and learning are based on the construction of hierarchical probabilistic models of the environment. These models are updated when top-down prior predictions are compared with incoming, sensory information, and the difference between the two (prediction error) is used to update the prior. Relative confidence in the prediction error and prior determines how much weight is afforded to each, and thus the extent to which beliefs are updated by incoming information versus prior knowledge. Bayesian and predictive coding accounts (referred to collectively as ‘Bayesian accounts’ from hereon^12,16,17^) propose that autism is characterised by atypical weighting of prior beliefs relative to incoming sensory information^11,16,18,19^. In support of this, research has demonstrated that autistic perception and learning are dominated by incoming sensory information, with less reliance on top-down priors^20–22^.

In principle, Bayesian accounts detail a general mechanism underpinning autistic processing which should apply to various domains of functioning. In the motor learning domain, the hypothesis that autistic individuals show underutilization of priors leads to specific and testable predictions. The motor system uses prior experience to prepare motor output for an event by an amount that is proportional to the probability of the event^23^. Thus, as the precision of an individual’s expectations about an upcoming action increases, reaction time (RT) decreases. However, if expectations are violated, RT increases (i.e., surprise-related slowing occurs) due to the requirement to halt the prepared action and prepare and execute the surprising action. Bayesian accounts of autism predict that the underutilisation of priors results in an aberrantly high baseline level of surprise. Thus, surprising events, which violate expectations, are not as surprising for autistic relative to non-autistic individuals^11,18^. According to such accounts, during motor learning surprise-related slowing should be reduced in autism (i.e. a more efficient response to surprising events should be observed), at the expense of learning a sequence and forming strong prior predictions about upcoming events^24^. To date, this has mainly been tested by demonstrating atypical surprise-related slowing with respect to *perception*. Lawson and colleagues^25^, for example, found that, relative to non-autistic controls, autistic adults showed reduced surprise in response to unexpected visual stimuli. It is currently not clear, however, whether Bayesian accounts of autism apply to motor learning. If so, they would help to shed light on the computational mechanisms underpinning differences in autistic motor learning.

Preliminary evidence for atypical surprise-related slowing in autism comes from several studies: Rinehart and colleagues^26^ required participants to execute button presses in response to a visual pattern, where surprising deviations from the pattern sporadically occurred. Relative to non-autistic children, autistic individuals showed a significant reduction in surprise-related slowing. Similarly, Gidley Larson and colleagues^27^, required participants to learn and execute a pattern of movements to anticipate the motion of a moving target*.* In several trials, expectations were violated by altering the pattern, inducing surprise-related slowing for non-autistic, but not for autistic, children. In line with Bayesian predictions, these studies suggest reduced surprise-related slowing in ASD. If underutilisation of priors is a pervasive style of autistic processing that cannot be unlearned or compensated for, establishing these effects in autistic adults is of central importance. However, to date, only one study has been carried out with adults, with results showing that participants were faster to respond to expected (referred to by the authors as validly cued) compared to unexpected (invalidly cued) events, there was no interaction between group (ASD versus control) and the validity of the cue type^28^. In sum, very few studies have investigated surprise-related slowing in ASD, and a coherent pattern of results has not emerged.

Extant studies of surprise-related slowing in autism have used paradigms with two response options (i.e. respond left or right^26^, trace circle or square^27^, change either hand or direction of movement^28^). A disadvantage of two-option paradigms is that, compared to multi-option paradigms, sequence-learning cannot be investigated. According to Hick’s Law^29^, the number of response options is logarithmically related to decision time, thus average RTs for a four-option paradigm in which each option is equally likely are 500–700 ms (ms), whereas a single response elicits an RT around 250–350 ms^30–32^. If, however, there is a sequence to the responses, one can reduce a four-option paradigm to a single response (with an associated probability) using prior knowledge of the sequence^8^, resulting in a greater RT reduction (i.e. a sequence-related speeding effect). The more potential options, the greater the potential speeding effect^33^. Since the extant autism literature has focused on two-option paradigms, it is not clear whether reduced surprise-related slowing (that is, a more efficient response to surprising events), comes at the expense of sequence-learning.

Here we compared the performance of autistic and non-autistic adults on a motor sequence-learning task^34^. Participants learned associations between four visual stimuli and four unique actions. In an ‘easy-predictable condition’, actions followed a simple sequence with occasional surprising trials where an unpredictable action was required. The same was true of the ‘difficult predictable condition’, although with a more challenging sequence. In the ‘unpredictable condition’ there was no sequence to learn. This task thus provides indices of sequence-learning, indexed by sequence-related speeding (the difference in RT between predictable and unpredictable conditions) and surprise-related slowing (the difference in RT between surprising and unsurprising trials in the predictable conditions). We predicted that (1) autistic adults would exhibit a *less* efficient response to unsurprising events, indexed by decreased sequence learning relative to non-autistic participants and (2) autistic adults would show a *more* efficient response to surprising events, indexed by a reduction in surprise-related slowing relative to non-autistic controls.

## Methods

### Participants

Twenty-eight adults with a clinical diagnosis of ASD (18–57 years, mean (standard deviation (SD)) age = 29.8 (10.2); 15 female), previously diagnosed by a UK National Health Service (NHS) or privately registered clinician who worked independently from our research group, according to the DSM^35^ or ICD-10^36^ criteria, and 35 healthy non-autistic controls (18–57 years, mean (SD) age = 27.6 (10.5); 13 female) were recruited from Birmingham and surrounding areas through advertising via posters and social media (see Table 1 for full demographic details and Supplementary Methods for full clinical details). All participants were reimbursed for their time (at a rate of £10 per hour) and travel expenses. ASD diagnosis was confirmed with administration of the Autism Diagnostic Observation Schedule, second edition ADOS-2^37^ by a trained researcher, using the current standard scores for a diagnosis of ASD, whereby a minimum score of 7 is the cut-off for designation as “on the autism spectrum”, and a minimum score of 10 is the cut-off for being designated as “autistic” (see Supplementary Methods for further inclusion criteria). The study was approved by the University of Birmingham local ethics committee (ERN_160281AP1R) and was conducted in accordance with the Declaration of Helsinki.

**Table 1:** Demographic information.

	Control group (n = 35) Mean (SD)	ASD group (n = 28) Mean (SD)	t (1,61)	*X*^2^ (1, *N* = 63)		p
Gender (*n *males: *n *females)	22:13	13:15		1.700		0.192
Age	27.6 (10.5)	29.8 (10.2)	1.496			0.140
Training trials	178.86 (177.16)	222.86 (133.19)	1.090			0.280
2-subscale IQ	107.51 (13.17)	108.679 (16.31)	0.314			0.755
Autism-Quotient (AQ)	15.09 (8.42)	36.46 (8.08)	10.196			< 0.001
Toronto Alexithymia Scale (TAS-2)	43.03 (10.67)	64.11(10.51)	7.842			< 0.001
ADOS total scores		7.64 (3.29)				

IQ was assessed with the Wechsler Abbreviated Scale of Intelligence-2 (WASI-2). SD refers to standard deviation. Training trials, IQ and gender did not significantly differ between the groups.

### General procedure

In a single session, participants first provided written, informed consent; second, completed the Autism-Quotient (AQ) questionnaire^38^, Toronto Alexithymia Scale (TAS-20^39^) and an Intelligence Quotient (IQ) test (two-item subscale of the Wechsler Abbreviated Scale of Intelligence—Second Edition WASI-II^40^), administered by a trained researcher (1 h); participants finally completed the serial reaction time task (1 h) followed by ADOS administration (1–2 h). All interviews were filmed for validation and training purposes.

### Serial reaction time task

**Figure 1:**
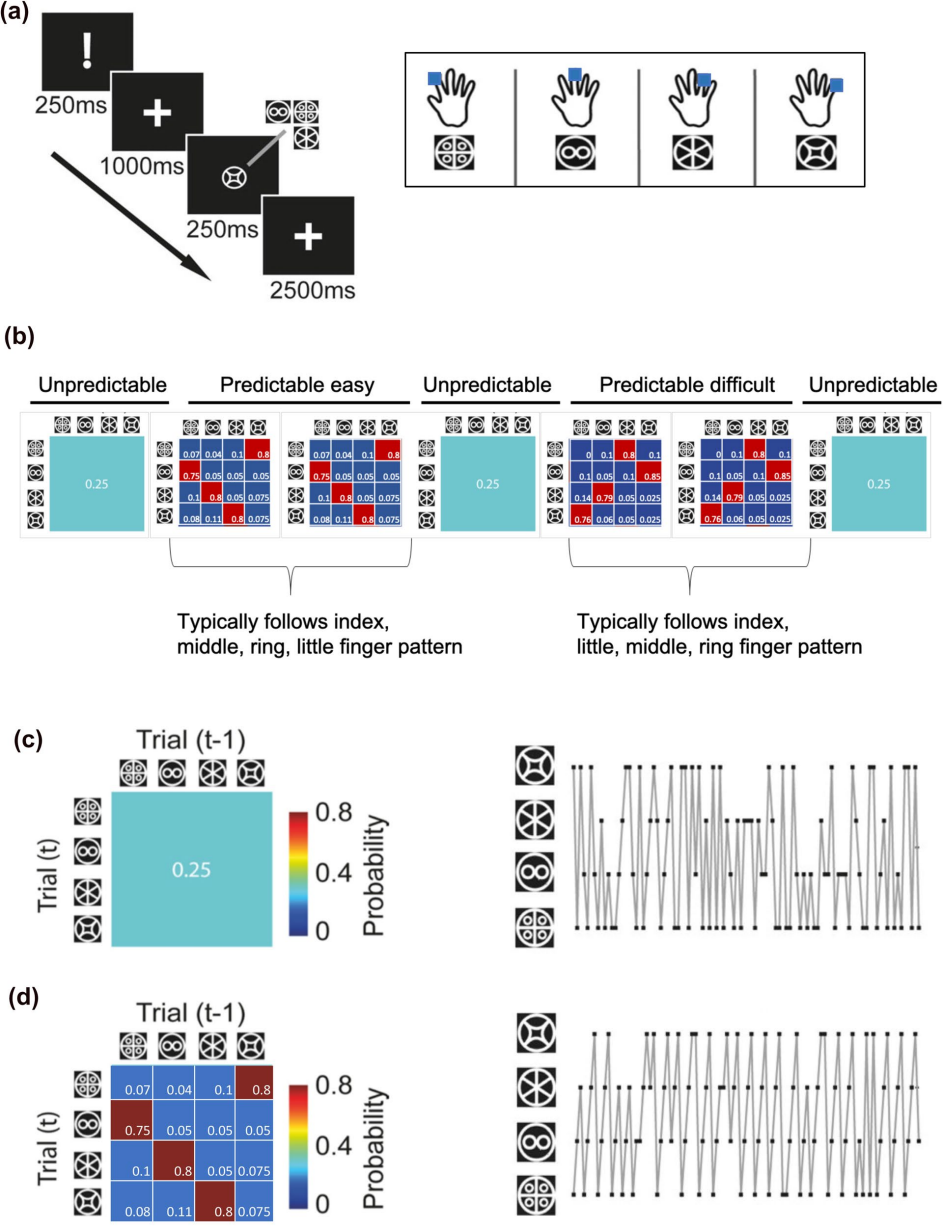
Behavioural task (**a**) Representation of a single trial. Participants observed a warning signal, followed by a fixation cross, then one of four different imperative stimuli (IS) and another fixation cross. Participants were advised to react as quickly as possible without sacrificing accuracy. Each of the four imperative stimuli corresponded to a specific finger press. (**b**) Overall task structure. Each participant completed seven blocks. Block order was the same for all participants. (**c**) Unpredictable condition. Transition matrix: All IS followed each other with equal probability, resulting in an unpredictable sequence. (**d**) Predictable condition. Sequences were generated from a first-order Markov sequence whereby numbers within the probability matrix represent the transition probabilities (16 possible combinations) and determined the relationship between the IS on trial *t* and trial *t-1*. The easy-predictable sequence is displayed here. Adapted from "Action reprogramming in Parkinson's disease: response to prediction error is modulated by levels of dopamine" by J. M. Galea et al., 2012, Journal of Neuroscience, 32(2):542–50. Copyright (2012) Galea et al.

Participants completed a probabilistic serial reaction time task, widely used to investigate motor sequence-learning^34,41^. Participants were instructed to place their index, middle, ring and little fingers on the keyboard letters V, B, N and M respectively. Subsequently, participants were required to learn associations between four imperative stimuli (IS) and four specific finger press actions (Fig. 1a) such that, for example, the appearance of a particular IS became associated with pressing the index finger down on the letter V. Participants were instructed to respond as quickly as possible to the IS. IS order followed different sequences depending on the condition, with predictable and unpredictable sequences presented in different blocks (Fig. 1b). A training period preceded the main experiment, in which participants learned associations between the IS and the specific actions. Subsequently participants completed seven blocks of 100 trials with self-paced rest intervals between the blocks. The task required approximately 45 min to complete in total. For the unpredictable condition, there was an equal probability of each IS appearing on each trial (Fig. 1c). For the easy-predictable condition, the sequence followed a pattern in which IS order 1–2–3–4 occurred with high probability (Fig. 1d). For the difficult-predictable condition, stimuli followed a more complicated pattern whereby the stimuli order 1–4–2–3 occurred with high probability. Surprising/unpredictable stimuli, which violated the sequence, occurred at a low probability, forcing participants to respond against their prior knowledge of the sequence, i.e., replace an expected action with an unexpected action. Surprising trials only occurred during the predictable blocks. The pattern or sequence in each block was not explicitly described. See Supplementary Methods for a more detailed task description and the participant instruction script.

### Statistical analysis

All analyses were conducted with MATLAB 2018b (MathWorks, Inc) and JASP (JASP Team, 2019). Raw and collated data and analysis scripts can be accessed at OSF (tiny.cc/58oxsz). RT was calculated as the time in milliseconds (ms) between onset of the IS and response (button press), for correct responses only. Since error rates and log-transformed error rates violated the assumptions for parametric testing (Levene’s test scores significantly differed from zero (p = 0.010)), Inverse Efficiency scores IES^42^ were instead used to account for possible speed-accuracy trade-offs. IES scores comprised the RT divided by 1 minus the proportion of correct responses (Supplementary Methods). To test whether autistic and non-autistic adults exhibit comparable sequence learning, RTs and IES were averaged for each condition and submitted to repeated-measures analysis of variance (RM-ANOVA), with the within-subject factor condition (easy-predictable, difficult-predictable and unpredictable) and between-subjects factor group (ASD, control). To test whether autistic and non-autistic adults exhibit comparable surprise-related slowing, RTs were calculated separately for surprising (probability > 0.75) and unsurprising (probability < 0.75) trials (Fig. 1d) separately for the predictable easy and difficult conditions and submitted to a RM-ANOVA with within-subject factors surprise (surprising, unsurprising), condition (easy-, difficult-predictable) and between-subjects factor group (ASD, control). To investigate whether the temporal evolution of surprise-related slowing differed between groups we modelled the effects of surprise on RT on a trial-by-trial basis. That is, alongside trial number and group, trial-by-trial surprise was included as a factor in a multiple regression analysis with RT as the dependent variable. The (trial specific) surprise of observing IS type *i* on trial *t* after experiencing IS type *j* on trial *t*-1 was calculated as the negative log of the IS pair’s (*ji*) predicted joint probability, with the joint probability of an IS pair on a given trial estimated from the number of previous occurrences of this IS pair on the preceding trials. Surprise thus represented the unexpectedness of the current IS (*i*) given the previous IS (*j*) based on the number of previous trials in which *j* preceded *i*^34^ (Supplementary Methods). Multiple regression was performed for each participant for each condition, with standardised β values then averaged across all participants within each group.

A threshold of p < 0.05 was used for all statistical tests, with significant effects investigated with Bonferroni-corrected post-hoc t-tests. For all analyses, Bayesian statistical testing was implemented as a supplement to null hypothesis significance tests. Bayes inclusion factors (BF_incl_) were included for all RM-ANOVAs, representing the evidence given the observed data for including a certain predictor in the model (see Supplementary Information for a full description of Bayesian analyses). For example, an inclusion Bayes factor for an effect of 3 for a given predictor *i* can be interpreted as stating that models which include the predictor *i* are 3 times more likely to describe the observed data than models without the predictor.

An a priori power analysis was carried out, based on research by Galea and colleagues^34^. Results showed that, based on an achieved effect size of η^2^p = 0.193, a minimum sample size of 22 participants per group was required. We complemented our a priori power analysis with post hoc sequential Bayesian testing. For our main effect of interest (surprise-related slowing), we continued data collection (and accumulation of evidence) until we had sufficient certainty about the absence of a group difference, i.e., the relative evidence for H0 plateaued above 3 (Suppl. Figure 1), representing moderate evidence for no group differences.

## Results

### Autistic and non-autistic adults exhibit comparable sequence learning

**Figure 2:**
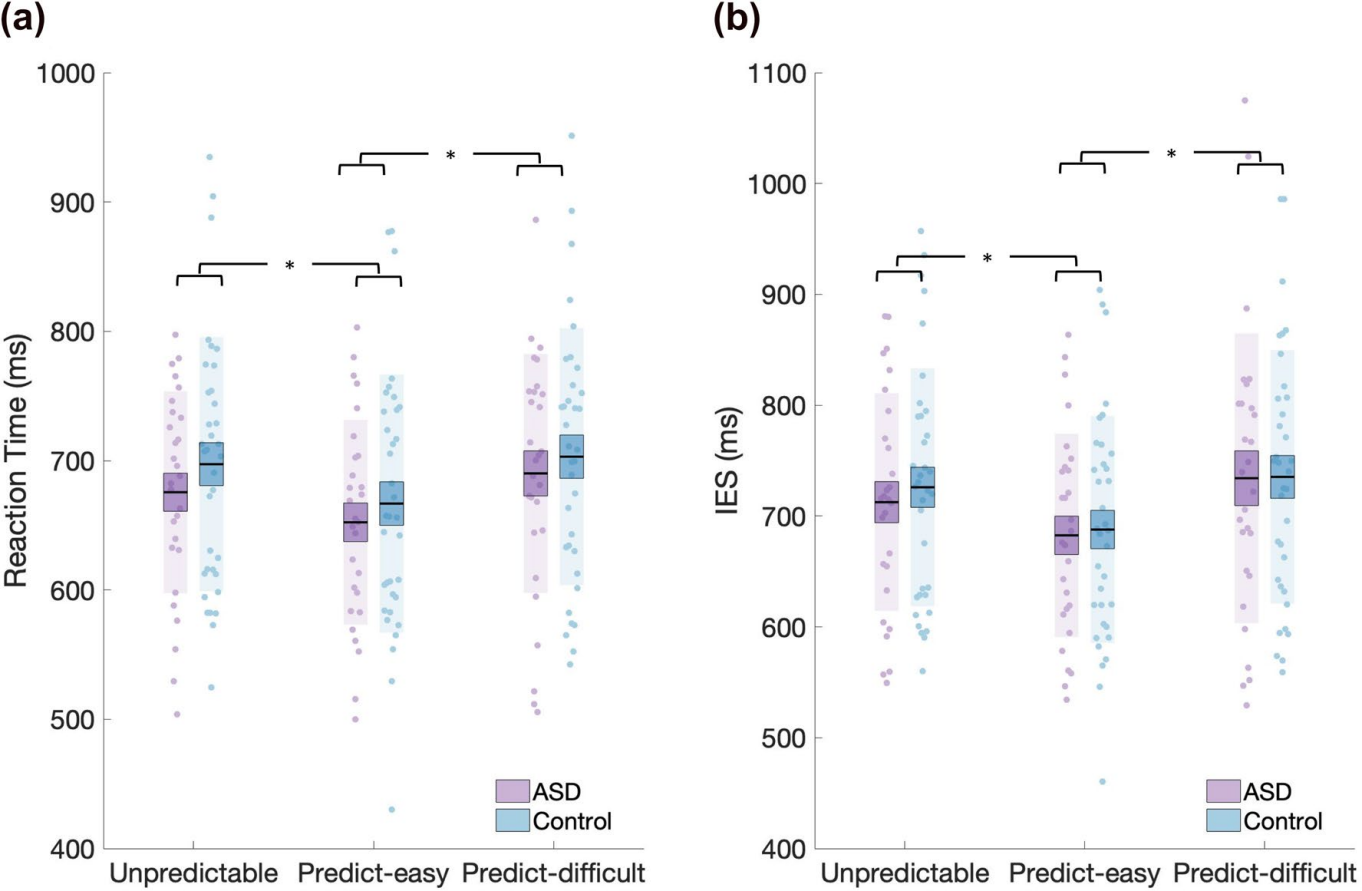
(**a**) Reaction time (RT). A significant difference in RT was observed between both the easy-predictable condition and the difficult-predictable and unpredictable conditions. ASD and control groups did not significantly differ in RT for any of the three conditions. (**b**) Inverse Efficiency Scores (IES). IES scores varied as a function of condition; no differences between groups were observed. Data points indicate individual participants. The mean is the thick black horizontal line and 1 standard error of the mean (SEM) is represented by the shaded box around the mean. Standard deviation (SD) is the shaded region.

**Figure 3:**
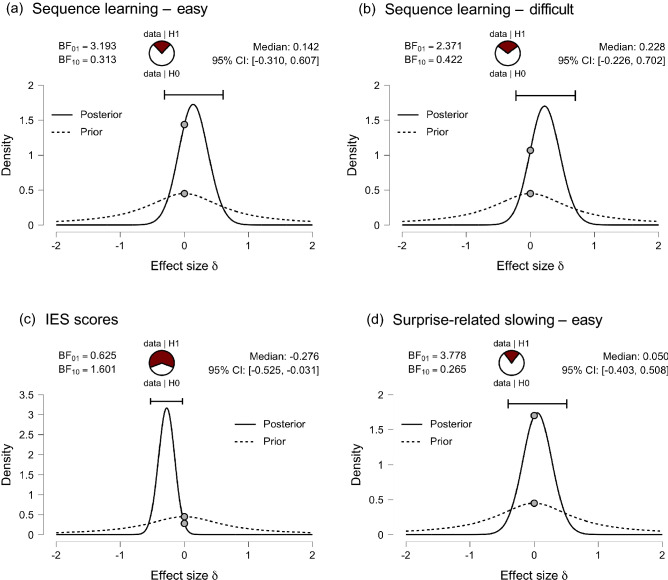
Bayesian statistical testing. The Bayes factor (BF_01_) indicates the evidence for the null hypothesis of no difference between groups. The density distribution displays the prior and posterior distribution for the population effect size, with the median effect size estimated, and a 95% credible interval which contains the median effect size. (**a**) Sequence learning – easy**:** BF_01_ = 3.193, meaning that the data are over three times more likely under H0 and provide moderate support for null hypothesis of no difference between groups (**b**) Sequence learning – difficult: BF_01_ = 2.371, meaning that the data are over two times more likely under H0 and provide anecdotal support for the null hypothesis of no difference between groups. (**c**) Bayesian paired t-test for IES scores in the predictable difficult condition compared with the unpredictable condition. BF_01_ = 0.625, meaning that the data are more likely under H1, providing weak support for the alternative hypothesis of a difference between conditions. (**d**) Surprise-related slowing – easy condition**.** BF_01_ = 3.778, meaning that the data are over three times more likely under H0 and provide moderate support for null hypothesis of no difference between groups.

To test our hypothesis that we would observe reduced sequence learning in the autistic group, as indexed by a reduced RT difference between predictable and unpredictable conditions relative to the control group, we submitted RTs to a RM-ANOVA with within-subject factor condition (easy-predictable, difficult-predictable, and unpredictable) and between-subjects factor group (ASD, control). This revealed a significant main effect of condition (F(2,122) = 28.804, p < 0.001, η^2^ = 0.028, BF_incl_ = 1.862e + 8) (Fig. 2a), no main effect of group (F(1,61) = 0.518, p = 0.474, η^2^ = 0.008, BF_incl_ = 0.423) and no interaction between condition and group (F(2,122) = 0.429, p = 0.652, η^2^ = 0.000, BF_incl_ = 0.182). Post-hoc Bonferroni-corrected t-tests demonstrated that RT was significantly lower for the predictable-easy (mean (standard error) $\overline{x } ({\sigma }_{\overline{x} })$ = 660.350 (11.438)) compared to the predictable-difficult ($\overline{x } ({\sigma }_{\overline{x} })$ = 697.318 (12.062), t(61) = − 6.707, p < 0.001, d = − 0.845) and unpredictable conditions ($\overline{x } ({\sigma }_{\overline{x} })$ = 687.624 (11.316), t(61) = -5.156, p < 0.001, d = − 0.65). Although RTs for the unpredictable and predictable-difficult conditions differed—with lower RTs for the unpredictable condition—this difference did not reach statistical significance (t(61) = 2.018, p_bonf_ = 0.137). In sum, we observed lower RTs for the predictable-easy compared to the unpredictable condition, suggesting that sequence learning enabled participants to speed their responses. However, we observed no significant speeding for the predictable-difficult condition, thus raising the possibility that the sequence was too challenging for participants to learn. Finally, the lack of a main effect of group suggested that the groups do not differ with respect to sequence-learning. This result was strengthened by Bayesian independent t-tests, with the BF indicating moderate evidence for H_0_ for the easy (BF_01_ = 3.193) and anecdotal evidence for the difficult (BF_01_ = 2.371) predictable conditions (Fig. 3a,b).

A RM-ANOVA with within-subject factor condition (easy-predictable, difficult-predictable and unpredictable) and between-subjects factor group (ASD, control) and IES as dependent variable revealed a main effect of condition (F(2,122) = 25.078, p < 0.001, η^2^ = 0.036, BF_incl_ = 1.585e + 7) and no main effect of group (F(1,61) = 0.064, p = 0.801, η^2^ = 0.001, BF_incl_ = 0.509) or group by condition interaction (F(2,122) = 0.377, p = 0.687, η^2^ = 0.001, BF_incl_ = 0.137) (Fig. 2b). Post-hoc tests demonstrated that IES were significantly lower for the predictable-easy ($\overline{x } ({\sigma }_{\overline{x} })$ = 685.502 (12.222)) compared to both the unpredictable ($\overline{x } ({\sigma }_{\overline{x} })$ = 719.985 (12.938), t(61) =  − 5.357, p < 0.001) and the predictable-difficult condition ($\overline{x } ({\sigma }_{\overline{x} })$ = 734.790 (15.226), t(61) =  − 5.942, p < 0.001). IES for the unpredictable compared to predictable difficult conditions did not significantly differ (t(61) =  − 2.155, p = 0.099, Cohen’s d = 0.271). However, a Bayesian paired t-test provided weak evidence that IES for the unpredictable blocks differed from the predictable difficult condition (BF_01_ = 0.625, Fig. 3c). Thus, RT, after correcting for number of errors, was higher during the predictable difficult, relative to the unpredictable, condition adding support for a lack of sequence learning during the difficult-predictable condition. The lack of a main effect of group, or interaction between group and condition suggests that the groups did not differ in the ability to execute the appropriate action. In sum, we did not find evidence to support the hypothesis that, relative to controls, autistic adults exhibited decreased sequence learning.

### Autistic and non-autistic adults exhibit comparable surprise-related slowing

**Figure 4:**
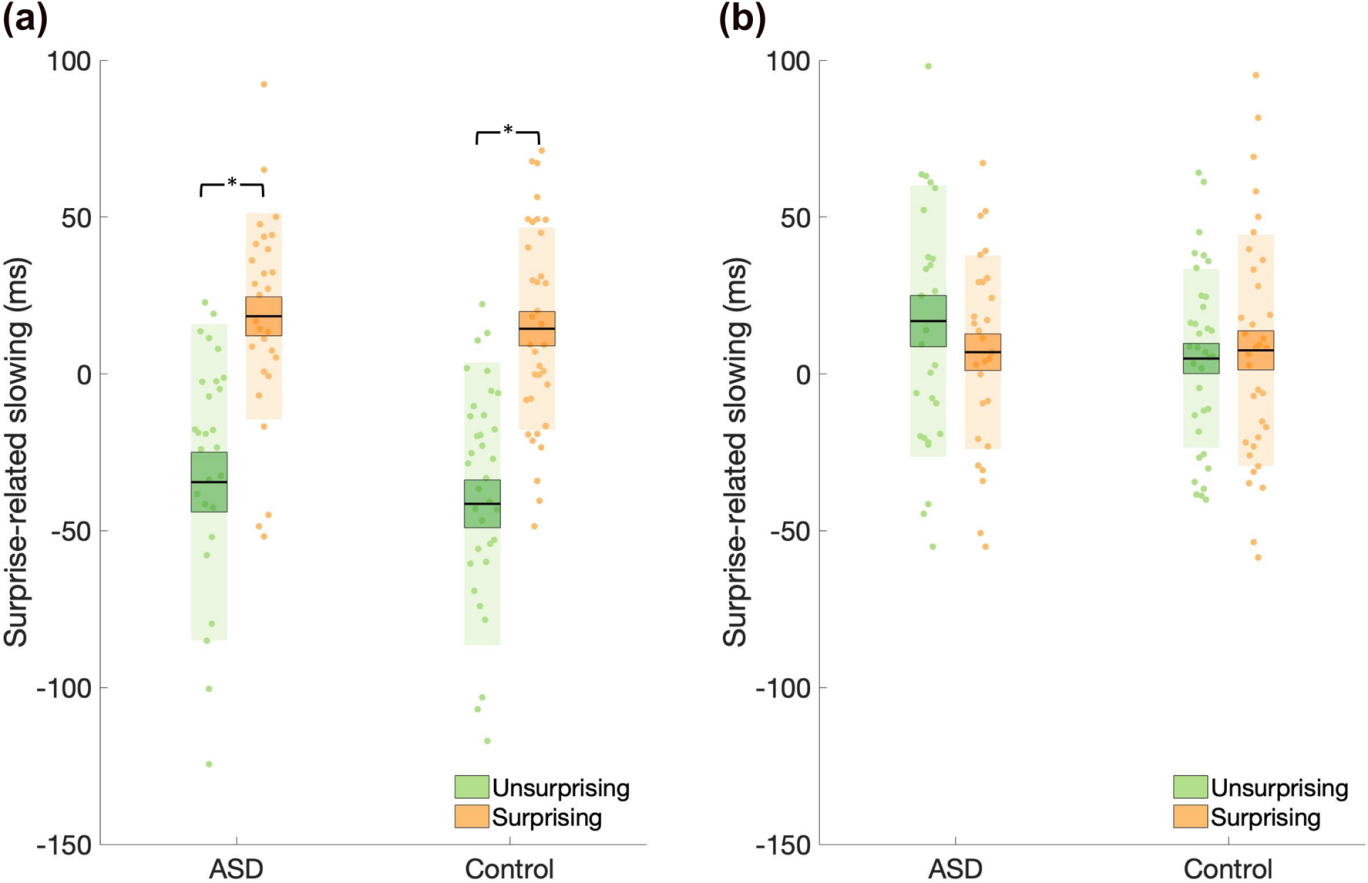
Surprise-related slowing (**a**) Easy-predictable condition**.** Data represent the difference between the mean reaction time (RT) for the unpredictable conditions and RT for surprising (orange) and unsurprising (green) trials. RT was significantly greater for surprising compared to unsurprising trials. (**b**) Difficult-predictable condition. There was no difference in RT between surprising and unsurprising trials in the difficult condition. No differences between the ASD and control group were observed in either condition. Data points indicate individual participants. The mean is the thick black horizontal line and 1 standard error of the mean (SEM) is represented by the shaded box around the mean. Standard deviation (SD) is the shaded region.

To test our second hypothesis that, relative to non-autistic controls, autistic participants would exhibit a reduction in surprise-related slowing—as indexed by a reduced RT difference between surprising and unsurprising trials—we submitted RTs to a RM-ANOVA with within-subject factors surprise (surprising trials, unsurprising trials), condition (easy-predictable, difficult-predictable) and between-subjects factor group (ASD, control). We observed main effects of surprise (F(1,61) = 34.144, p < 0.001, η^2^ = 0.017, BF_incl_ = 1.166e + 13), condition (F(1,61) = 34.144, p < 0.001, η^2^ = 0.017, BF_incl_ = 4.526e + 13) and a surprise by condition interaction (F(1,61) = 72.325, p < 0.001, η^2^ = 0.022, BF_incl_ = 3.714e + 8). Post-hoc comparisons revealed an increase in RT for surprising compared to unsurprising trials for the easy-predictable (surprising: $\overline{x } ({\sigma }_{\overline{x} })$ = 703.782 (11.744), unsurprising: $\overline{x } ({\sigma }_{\overline{x} })$ = 649.278 (11.575), mean difference = 54.340; t(61) = 9.850, p < 0.001, d = 1.241) (Fig. 4a) but not the difficult-predictable condition (surprising: $\overline{x } ({\sigma }_{\overline{x} })$ = 694.875 (12.011), unsurprising: $\overline{x } ({\sigma }_{\overline{x} })$= 697.835 (12.241), mean difference = − 3.656; t(61) =  − 0.663, p = 1.000, d = − 0.083) (Fig. 4b). Crucially, no main effect of group (F(1,61) = 0.493, p = 0.485, η^2^ = 0.008, BF_incl_ = 0.326), surprise by group (F(1,61) = 0.797, p = 0.375, η^2^ = 0.000, BF_incl_ = 0.267), condition by group (F(1,61) = 0.795, p = 0.980, η^2^ = 0.000, BF_incl_ = 0.199) or surprise by condition by group (F(1,61) = 0.493, p = 0.485, η^2^ = 0.000, BF_incl_ = 0.088) interactions were observed. To ensure that the lack of group difference could not be attributed to differences in baseline speed, we re-ran the analysis with baseline-corrected mean RT scores. This did not change the observed pattern of results, with no main/interaction effect(s) of group observed (all p-values > 0.05, all η^2^ < 0.001, all BF_incl_ < 1). Indeed, no differences in motor execution overall were observed between groups (Supplementary Results).

Surprise-related slowing scores for the two groups in the easy-predictable condition were compared using a Bayesian independent t-test. The BF_01_ was equal to 3.778, indicating the data were approximately 3.8 times more likely under the hypothesis that groups did not differ with respect to surprise-related slowing and providing moderate evidence for H_0_ (Fig. 3d).

Finally, although IES scores varied significantly across condition (F(1,61) = 16.538, p < 0.001, η^2^ = 0.011, BF_incl_ = 106.780), surprise (F(1,61) = 43.141, p < 0.001, η^2^ = 0.031, BF_incl_ = 1.905e + 7) and condition by surprise (F(1,61) = 56.538, p < 0.001, η^2^ = 0.026, BF_incl_ = 2.649e + 7), no main/interaction effect(s) of group were observed (all p-values > 0.05, all η^2^ < 0.001, all BF_incl_ < 1) (Suppl. Figures 2a-2b).

### Trial-by-trial surprise did not differ between groups

The observed results demonstrated typical surprise-related slowing in autistic individuals. However, the above analyses collapse data across all trials within each condition and cannot detect differences in the temporal progression of surprise-related slowing, nor reveal differences between the groups in the speed of acquisition of surprise-related slowing. Trial-by-trial surprise was therefore included as a predictor in a multiple regression analysis, alongside trial number and condition. Standardized beta values (β) for the main and interaction effect(s) of predictors (Table 2) were compared using one-sample t-tests to determine if they were significant predictors of RT. β values that significantly differed from zero were averaged across each group and compared using standard and Bayesian independent sample t-tests. If differences in the temporal progression of surprise-related slowing existed between groups, we would expect to observe a significant difference in β values relating to the interaction between surprise and trial number. However, no differences in β values were observed between groups for this interaction (t(61) = 1.130, p = 0.263, d = 0.287, BF_01_ = 2.260), nor for β values for condition (t(61) =  − 0.022, p = 0.983, d = − 0.005, BF_01_ = 3.868), trial-by-trial surprise (t(61) = 0.905, p = 0.369, d = 0.229, BF_01_ = 2.739) or surprise by condition (t(61) = 1.191, p = 0.238, d = 0.302, BF_01_ = 2.130). In summary, no differences in the temporal evolution of surprise-related slowing were observed between groups.

**Table 2:** Predictors of reaction time.

	Standardized β values	Standard Error β values	t(62)	p	Cohen’s d
Time	1.430	1.776	0.805	0.424	0.101
Condition	18.793	2.821	6.663	< 0.001***	0.839
Surprise	9.707	1.598	6.076	< 0.001***	0.766
Time × condition	0.026	2.247	0.012	0.991	0.001
Time × surprise	1.116	1.120	0.996	0.323	0.126
Condition × surprise	− 10.432	1.414	− 7.3766	< 0.001***	− 0.929
Time × condition × surprise	1.612	1.300	1.239	0.220	0.156

Standardized beta values (β) are displayed for significant and non-significant predictors of reaction time.

### ADOS severity scores as a predictor of surprise-related slowing and sequence-learning

Focusing specifically on the easy-predictable condition, where surprise-related slowing and sequence-learning effects were observed, correlation analysis showed that ADOS scores were not a significant predictor of surprise-related slowing (r = − 0.324, F(1,26) = 3.042, *p* = 0.093) or sequence-learning (r = 0.070, F(1, 26) = 0.129, *p* = 0.723). Furthermore, neither AQ nor TAS scores were significant predictors of behavioural measures (Supplementary Results).

## Discussion

Here we investigated the underutilisation of priors in ASD in the context of a motor sequence-learning task. In predictable conditions, actions largely followed a pre-defined sequence with infrequent surprising violations of this sequence. In the unpredictable condition, there was no sequence to learn. In line with Bayesian accounts of autism, we hypothesised that autistic adults would show a more efficient response to surprising events at a cost to sequence learning, indexed by a reduction, relative to non-autistic controls, in surprise-related slowing alongside decreased sequence-learning. Contrary to our predictions, there were no significant differences between autistic and non-autistic adults in terms of surprise-related slowing or sequence learning. Furthermore, Bayesian statistics provided anecdotal to moderate evidence to support the conclusion that the groups were comparable with respect to both measures.

The lack of a difference between the groups departs from the predictions of Bayesian accounts of autism. One potential explanation for this conflict is that our sample might not be representative of the populations typically used to test these accounts. In opposition to this, we argue that our sample is comparable in terms of age, IQ and average ADOS score to a number of studies that have found evidence in support of the underutilization of priors in ASD^25,43–45^. Thus, suggesting that the level of autism symptomatology, age or IQ of our participants is unlikely to explain the observed null results, though we note that comparison between studies is challenging due to the use of different paradigms. In addition, Bayesian analyses revealed that we had anecdotal evidence to support the null hypothesis that there is no correlation between ADOS scores and the extent of surprise-related slowing. This suggests that recruiting a more diverse sample is unlikely to alter the observed results. Indeed, if the relationship between ADOS and surprise-related slowing is linear, we would not expect different results with a broader sample. Nevertheless, we acknowledge that this claim requires empirical testing since at present we have only anecdotal evidence for the lack of a relationship between ADOS and surprise-related slowing and, furthermore, it is possible that a non-linear relationship exists (e.g., there could be a step change in surprise-related slowing with increasing ADOS score). Consequently, we can confidently conclude, based on our Bayesian and frequentist analyses that in our sample (with age, AQ and ADOS ranges of 18–57, 17–48 and 2–14 respectively) there is no evidence of underutilisation of priors. Further empirical testing would be necessary to be confident that this conclusion extends to samples of the autistic population with different characteristics.

A further potential explanation for the conflict between the current results and the predictions from Bayesian accounts of autism is that our task does not really index the process of evaluating and updating priors predicted by Bayesian accounts of autism. The motor sequence featured in the easy-predictable condition is easily executable and could potentially be explicit in nature. Some authors have argued that explicit motor learning relies less on priors and prediction errors and more on target-driven error derived from an explicit strategy, although results thus far derive from sensorimotor adaptation tasks^46,47^. However, the current paradigm employed a probabilistic sequence learning structure, frequently used as a measure of *implicit* learning, whereby a predictable sequence is frequently interspersed with “surprising” stimuli^48^. Such surprising trials decrease explicit knowledge of the sequence in comparison to a fixed or deterministic sequence^49,50^. Additionally, Galea and colleagues^34,41^, demonstrated that the (dopamine-dependent) prediction error process is central to this task, observing increased surprise-related slowing in the context of the same motor sequence learning task in adults with Parkinson’s disease when off- compared to on-medication^34^ and under dopamine antagonism in healthy adults^41^. Consequently, we believe that our task provides a good measure of the utilisation of priors.

One might ask whether the logical conclusion from our results is that Bayesian accounts of autism do not apply in the motor domain. Indeed, research relating to Bayesian accounts of autism has primarily focused on sensory/perceptual processing^20–22,51,52^, leading to the possibility that Bayesian accounts of autism are restricted to these domains. However, Bayesian accounts have been proposed as a general principle of information processing across various domains including motor function^53^. Furthermore, attenuated priors have been suggested to account for difficulties in movement preparation and planning in autism^11^ and reduced slowing for unexpected movements has been demonstrated for autistic children relative to controls^26,27^. Thus, there is little reason to believe that Bayesian accounts would be restricted to the sensory/perceptual domain.

This lack of a theoretical or empirical basis to support the conclusion that Bayesian accounts of autism do not extend to the motor domain forces us to consider alternative explanations. For example, our results clearly contrast with recent research showing attenuated surprise-related slowing (albeit in the context of learning auditory-visual as opposed to motor-motor associations) in autistic adults^25^. However, a notable difference between the current paradigm and the one employed by Lawson and colleagues is that the latter concerned a learning environment containing multiple levels of uncertainty including, probabilistic uncertainty (i.e. the auditory stimulus could be weakly, strongly or not at all predictive of the visual stimulus) and crucially, variation in the uncertainty of the learning environment itself (i.e. “environmental volatility”) such that some periods featured frequent reversals in learned associations, and others rarely featured reversals. Lawson and colleagues argue that group differences in surprise-related slowing stem from an overestimation of environmental volatility. Results were in accordance with recent updates to predictive coding accounts of autism, which propose that, while general learning is unaffected, meta-learning (learning about learning, as when one learns about the statistics (e.g. volatility) of a learning environment), is atypical^11,18,54^. The current paradigm contained only probabilistic uncertainty (i.e., the current action could be weakly or strongly predictive of the action in the subsequent trial), with no requirement to learn higher-order statistics about the environment. Therefore, it is possible that our task did not tap into the (meta-learning related) predictive processes that are thought to be a key point of difference between autistic and non-autistic individuals. Work from Manning and colleagues^55^, however, casts doubt on this potential explanation. Using a probabilistic reward-learning paradigm which demanded learning of environmental volatility, Manning and colleagues demonstrated that autistic children successfully adapted their learning rate to suit the level of environmental volatility. To investigate whether the current (null) results are due to a lack of variation in environmental uncertainty, an adapted version of our paradigm that demands learning about environmental volatility—such as that developed by Marshall et al.^56^—could be employed.

Finally, contrasting findings could be related to different networks of brain regions recruited across different tasks. Sequence learning tasks have relatively low motor demands and do not require the acquisition of a novel movement, thus they predominantly rely on connections within the motor cortical and subcortical regions^57^. In contrast, several tasks in which performance is atypical in ASD require integration between distinct brain regions^25,44^ and thus rely on long-range connectivity. Neuroimaging studies have linked autism to alterations in the coordinated activity of distant brain regions^58–60^. Indeed, autism has been associated with underconnectivity for long-range cortico-cortical connections^61,62^ and theoretical accounts have linked this to the underutilisation of priors^63,64^. Furthermore, with respect to motor function, Gowen and Hamilton^65^ have argued that motor learning per se is not atypical in autism, however, complications arise when cross-modal integration, which relies on long-range connectivity, is required. It is, therefore, possible that the influence of top-down priors is predominantly attenuated in tasks that rely on long-range connectivity between cortical regions^25,66,67^.

In summary, after considering both frequentist and Bayesian statistics, we did not find evidence for differences in surprise-related slowing or sequence learning between autistic and non-autistic adults in a motor sequence learning task. Our results fail to provide evidence in support of straightforward predictions from Bayesian accounts of autism in the context of motor learning. Consequently, these data highlight that more nuanced Bayesian accounts of autism (potentially considering the role of factors such as meta-learning or long-range connectivity demands) are required if such accounts are to be extended to the domain of motor learning.

## Supplementary Information

Supplementary Information.

## Data Availability

Raw and collated data and analysis scripts used in the current study can be accessed from an Open Science Framework (OSF) repository (tiny.cc/58oxsz).
