# Supplementary material for: Intact predictive motor sequence learning in autism spectrum disorder
Source: Sci Rep. 2021 Oct 19;11:20693. doi: 10.1038/s41598-021-00173-1 (PMC8526822; doi:10.1038/s41598-021-00173-1)
Supplement: Supplementary file 1 — Supplementary Information. [file 41598_2021_173_MOESM1_ESM.docx]

Supplementary information

Intact predictive motor sequence learning in autism spectrum disorder

Rybicki, A. J.*, Galea, J. M., Schuster, B. A., Hiles, C., Fabian, C., & Cook, J. L.

[Methods 2](#_Toc80207816)

[Autism spectrum group clinical information 2](#_Toc80207817)

[Serial reaction time behavioural task 2](#_Toc80207818)

[Trial-by-trial surprise 4](#_Toc80207819)

[Inverse Efficiency scores 5](#_Toc80207820)

[Bayesian statistical testing 5](#_Toc80207821)

[Results 7](#_Toc80207822)

[AQ and TAS scores as a predictor of surprise-related slowing 7](#_Toc80207823)

[Comorbidity and Medication 7](#_Toc80207824)

[Motor execution 7](#_Toc80207825)

[Fig. S1 9](#_Toc80207826)

[Fig. S2 10](#_Toc80207827)

[Tables 11](#_Toc80207828)

[References 12](#_Toc80207829)

# Methods

Participant inclusion criteria

All participants were fluent English speakers, with no history of neurological disease, muscular dystrophy or cerebral palsy, and had an intelligence quotient (IQ) score, measured using the two-subscale measure of the Wechsler Abbreviated Scale of Intelligence- 2 (WASI-2; Wechsler, D., 2011), greater than 75.

## Autism spectrum group clinical information

Sixteen (57%) of the participants reported direct family history of developmental, neurological or severe psychiatric disorders. Six subjects had an additional diagnosis of a psychiatric disorder: one with bipolar disorder, one with epilepsy, two with attention deficit disorder (ADHD), three with generalized anxiety disorder and two with major depression. Sixteen (57%) of the sample were on prescribed psychiatric medication, with antidepressants the most commonly prescribed medication (twelve subjects), followed by ADHD medication (three subjects) and epilepsy (one subject). Six subjects (21%) had a learning disorder and two (7%) reported developmental delay. 2-subscale IQ scores ranged from 78-142 (mean (SD) = 108.68 (16.31) All analyses were repeated with the exclusion of sub-groups based on clinical information (see Supplementary results).

## Serial reaction time behavioural task

Participants completed a computerised motor sequence learning task run on MATLAB 2018b (MathWorks, Inc.; http://www.mathworks.co.uk/) with Cogent 2000 (http://www.vislab.ucl.ac.uk/cogent_2000) for which they sat in front of a computer screen (approx. 30 cm distance from screen), with the keyboard placed in front of their dominant hand. They were instructed to place each of their fingers separately on the keyboard letters V, B, N and M and to maintain this position throughout the task. A Razer DeathStalker keyboard, with ultra-polling at 1000Hz enabled, was used for precise measurement of RT.

For each individual trial, the following events were presented to the participant: A warning cue was displayed for 250 milliseconds (ms), followed by a fixation cross (1000 ms). After this, one of four imperative stimuli (IS) were shown in the centre of the screen for 250 ms (Fig. 1a). During this time, the participant was required to respond to the displayed stimuli. Stimulus presentation was followed by the fixation cross (2500 ms). Each IS was associated with a specific keyboard key, which corresponded to a specific finger press action (V, B, N, or M). Participants were required to learn the association between the IS and action (e.g., middle finger press) and execute the associated action when presented with an IS. Participants were instructed to respond as fast as possible, without sacrificing accuracy. Participants had to complete at least two training blocks of 60 trials in which they scored over 90% accuracy, before they could progress to the main experiment. If performance on the second training block was less than 90% correct, another block was added, this procedure was repeated until performance exceeded 90% correct. During the training blocks, participants received textual on-screen feedback as to whether their response was correct (“Correct!!!”) or incorrect (“Wrong”). During training, stimuli were presented in an unpredictable order, with equal probabilities (25%) of each stimulus appearing on each trial. After the training round, they were asked to respond as quickly and accurately as possible to the presented symbols. They were shown the following instructions on the computer screen before starting the task:

*“The experiment will start soon. Please remember to stay focused all the time! Please get ready when you see the warning cue, this will allow you to respond fast and accurately”*

In the main experiment, participants completed seven blocks of 100 trials with self-paced rest intervals between the blocks. Feedback was not given in the main experiment. In the main experiment, IS order followed different sequences depending on condition, with predictable and unpredictable sequences presented in different blocks (Fig. 1b). For blocks one, four and seven (Fig. 1c) the stimulus sequence was unpredictable, with an equal probability (0.25) of each stimulus appearing on each trial. For blocks two, three, five and six, stimulus presentation followed a predictable pattern (see Fig. 1d for the easy predictable probabilities). Each stimulus was drawn from a predictable first-order Markov sequence, where the current stimulus t was dependent on the stimulus presented at the previous trial, t-1. Therefore, predictable sequences were generated by sampling from a distribution specified in a transition matrix which quantified the dependence between stimuli. For the first predictable condition (Fig. 1d), the sequence followed an easy pattern in which IS order 1-2-3-4 (corresponding to the keyboard presses V-B-N-M) occurred with high probability, requiring the participant to respond with the natural order of the fingers, i.e., index, middle, ring and little finger. For the difficult-predictable condition stimuli followed a less natural predictable pattern whereby the stimuli order 1-4-2-3 (V-M-B-N) occurred with high probability.

## Trial-by-trial surprise

Surprise was quantified on a trial-by-trial basis in a stimulus-specific manner, with the surprise (S) of observing a stimulus *i* on trial *t* after experiencing stimulus type *j* on trial *t*-1 calculated using the assumption that subjects behaved as “ideal” observers, beginning each block with the prior expectation of all stimulus pairings being equally probable, and updating the conditional probability of each pairing using a Bayesian update scheme [1],[2]. On each trial (*t*), subjects were presented with one of four IS, with the conditional probability of an IS on a given trial estimated from the previous occurrences of IS on the preceding trials. Specifically, the conditional probability (*E*) of an IS ﻿at trial *t*, *p*(*E_t_*), was estimated from the number of occurrences of IS *i* up to trial *t* ($n_{i}^{t}$, where *i* indexes the IS type and *t* the trial number) (Equation 1). Thus, the estimate of *i* at a given trial *t* was defined by:

$p_{t}\left( E_{t}=i \right)=\frac{n_{i}^{t}+1}{\sum_{i} (n_{i}^{t}+1)}, (p_{0}{E_{0}=i)= \frac{1}{4}}$ (Equation 1)

Due to the probabilistic structure of the first-order Markov sequence, the IS occurring on the previous trial (*t*-1) could be used to form the prediction for the IS on trial *t*, allowing an approximation of the joint probability distribution for each IS pair to be estimated from the count of previous occurrences of the IS pair up to trial *t* ($n_{ij,}^{t}$ *i* = current IS type and *j* = previous IS type, Equation 2).

$p_{t}\left( E_{t}=i, E_{t-1}=j \right)= \frac{n_{ij}^{t}+1}{\sum_{i,j} (n_{ij}^{t}+1)}$ (Equation 2)

The surprise (*S*) of observing IS type *i* on trial *t* after experiencing IS type *j* on trial *t*-1 was therefore calculated as the negative log of the IS pair’s predicted joint probability (Equation 3).

$S\left( E_{t}=i, E_{t-1}=j \right)= -log 2\left( p\left( E_{t}=i, E_{t-1}=j \right) \right)$ (Equation 3)

Surprise was therefore stimulus-specific, representing the unexpectedness of the current IS, given the IS at trial *t*-1 and was high when an IS pairing was infrequent and low when the paring was frequent or occurred at a high probability (Fig. S1). Surprise was low overall during the predictable sequences, with occasional violations when unlikely surprising IS pairs occurred. In the unpredictable blocks, all events were equally as surprising as stimulus-pair probabilities were all fixed at 0.25.

## Inverse Efficiency scores

Due to the extensive practice period, error rate was low across all conditions. Further to this, reaction time and accuracy were not independent in this task, as participants were required to respond within a narrow time frame. As this could lead to a speed-accuracy trade-off, whereby variable measures could lead to contradictory conclusions about the effect of group, we included a measure which combined speed (inverse RT) and accuracy. The inverse efficiency score (IES) [3] was utilized. This measure divided RT by 1 minus the proportion of errors (PE) or the proportion of correct responses (Equation 4). IES scores were calculated for each condition and for surprising and unsurprising trials separately.

$$IES=\frac{RT}{(1 -PE)}$$

(Equation 4)

## Bayesian statistical testing

Bayesian statistical testing was implemented as a supplement to null hypothesis significance tests, to compare the likelihood of the data under the null and alternative hypothesis and provide an estimate for the amount of evidence represented in the data, in order to investigate if null results represent a true lack of a difference between the groups [4]. Bayesian statistical testing was implemented in JASP, based on the R package “BayesFactor” [5].

A Bayesian t-test framework [6] was used to determine if there was a difference in surprise-related slowing between the groups, for both the easy and difficult conditions. A Bayes factor, comparing the fit of data under the null hypothesis and the alternative hypothesis was estimated [7],[8] for each condition, whereby the null hypothesis (H_0_) postulates that there are no differences between groups for surprise-related slowing scores. A two-sided alternative hypothesis was used, allowing the effect size (δ) to take both positive and negative values, with a default Cauchy prior distribution for a two-sample t-test, specifically, a zero-centred Cauchy distribution with a scale of 0.707 [7].

Bayesian repeated measures ANOVAs were utilized to investigate the effects of surprise, condition and group on RT and IES scores. The JASP framework for repeated measures ANOVA was used [9], whereby candidate models M and their condition-effect parameters β were compared, resulting in Bayes factors for each candidate model, quantifying the relative predictive performance of different models, as well as inclusion Bayes factors for predictors of interest. The inclusion Bayes factor (BF_incl_) for a given predictor quantifies the change in odds from the prior probability that the predictor is included in the model to the probability of inclusion in the model after seeing the data, and were computed by comparing all models with a predictor against all models without that predictor, i.e., comparing models that contain the effect of interest to equivalent models stripped of the effect. For example, an inclusion Bayes factor for an effect of 3 for a given predictor *i* can be interpreted as stating that models which include the predictor *i* are 3 times more likely to describe the observed data than models without the predictor. In short, the inclusion Bayes factor is interpreted as the evidence given the observed data for including a certain predictor in the model. The inverse of this, the Bayes exclusion factor (BF_excl_) therefore represents the evidence for excluding a certain predictor. For example, a BF_excl_ value of 3 for a given predictor *j* means that models that *exclude* the predictor *j* are 3 times more likely given the data than models which include it. For all Bayesian analyses, the Bayes factor quantifies the relative evidence for one theory or model over another. We followed the classification scheme used in JASP [10] to classify the strength of evidence given by the Bayes factors, with BF_01_ between one and three considered as weak evidence, between three and ten as moderate evidence and greater than ten as strong evidence for the null hypothesis respectively. An annotated .jasp file containing all analysis is available at https://osf.io/cax4g/.

# Results

## AQ and TAS scores as a predictor of surprise-related slowing

As AQ and TAS scores differed significantly between the groups, we conducted separate linear regression analyses to investigate the potential effect of AQ and TAS score severity on surprise-related slowing. Results demonstrated that neither AQ (β = 0.031, R^2^ = 0.001, *t*(62) = 0.241, *p* = 0.810) nor TAS scores (β = 0.019, R^2^ = 0.000, *t*(62) = 0.147, *p* = 0.884) predicted the extent of surprise-related slowing. Repeated the above analyses with sequence learning as the dependent variable yielded similar results: neither AQ (β = 0.139, R^2^ = 0.019, *t*(62) = 1.096, *p* = 0.277) nor TAS scores (β = 0.115, R^2^ = 0.115, *t*(62) = 0.900, *p* = 0.371) predicted the extent of sequence learning.

## Comorbidity and Medication

We re-ran all analyses with the exclusion of participants with current psychiatric medication use (n = 16). There were no significant differences between for any of the main measures of interest between the full sample and the sample excluding medication use, including sequence learning (all main/interaction effect(s) of group: all p values > 0.05, all η² <0.01) and surprise-related slowing (all main/interaction effect(s) of group: all p values > 0.05, all η² <0.01). Repeating the analyses with the exclusion of participants with comorbid psychiatric conditions (n = 7) yielded similar results (all main/interaction effect(s) of group: all *p* values > 0.05, all η² <0.01).

## Motor execution

Performance in the unpredictable blocks enable us to obtain a measure of participants’ motor ability, as, since there are no sequences to learn in the unpredictable blocks, any differences in speed/error are likely to reflect motor execution ability. No significant differences were found between CTRL and ASD in RT (unpaired t-test: t(61) = 0.951, p = 0.345, d = 0.241, BF_01_ = 2.642) or IES (unpaired t-test: t(61) = 0.513, p = 0.610, d = 0.130, BF_01_ = 3.462) when we examined unpredictable trials (averaged across 3 blocks). Bayesian independent samples t-tests supported these results, with BF_01_ = 2.642 providing anecdotal evidence for no difference in RT between groups, and BF_01_ = 3.462 providing moderate evidence for no difference in IES scores between groups. We then investigated whether motor execution (RT/IES in unpredictable blocks) was correlated with other measures. No correlation was found between RT in the unpredictable blocks and sequence-learning in either the easy (r = 0.211, p = 0.098) or difficult (r = -0.012, p = 0.924) predictable blocks. In addition, there was no correlation between RT in the unpredictable blocks and the extent of surprise-related slowing in either the easy (r = 0.164, p = 0.200) or difficult (r = -0.032, p = 0.805) predictable blocks, suggesting that our measures (surprise-related slowing and sequence learning) are independent of motor execution.

# Fig. S1


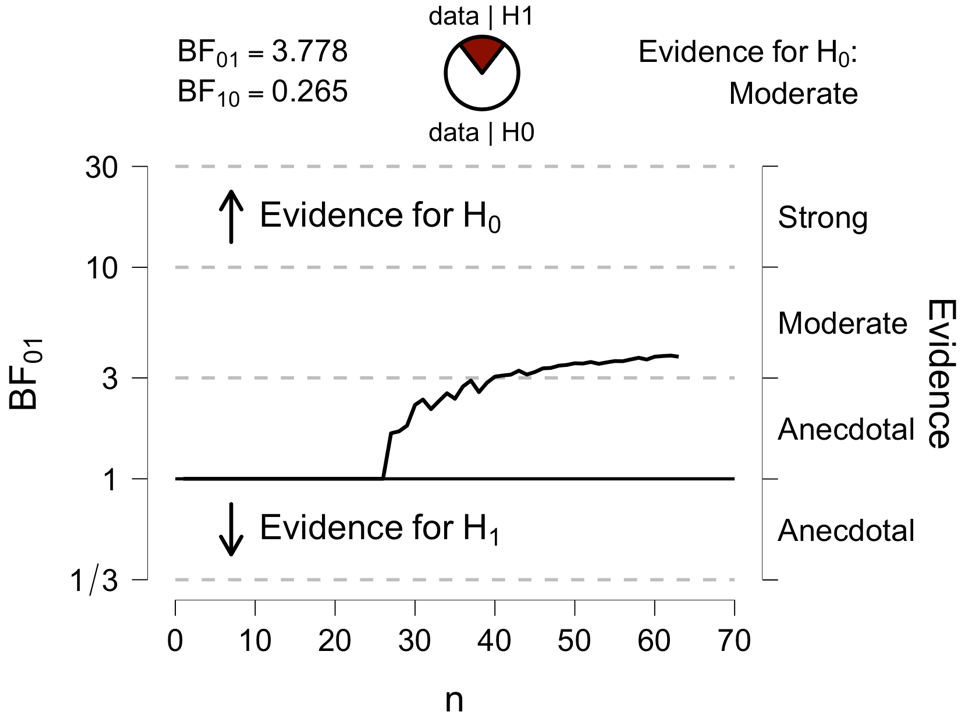


**Fig. S1. Sequential analysis.** Sequential analysis represents a visualisation of evidence as data are collected.

The black line represents accumulation of evidence for the null hypothesis (BF_01_) that groups do not differ in the extent of surprise-related slowing during the easy-predictable condition.

# Fig. S2

(b)

(a)


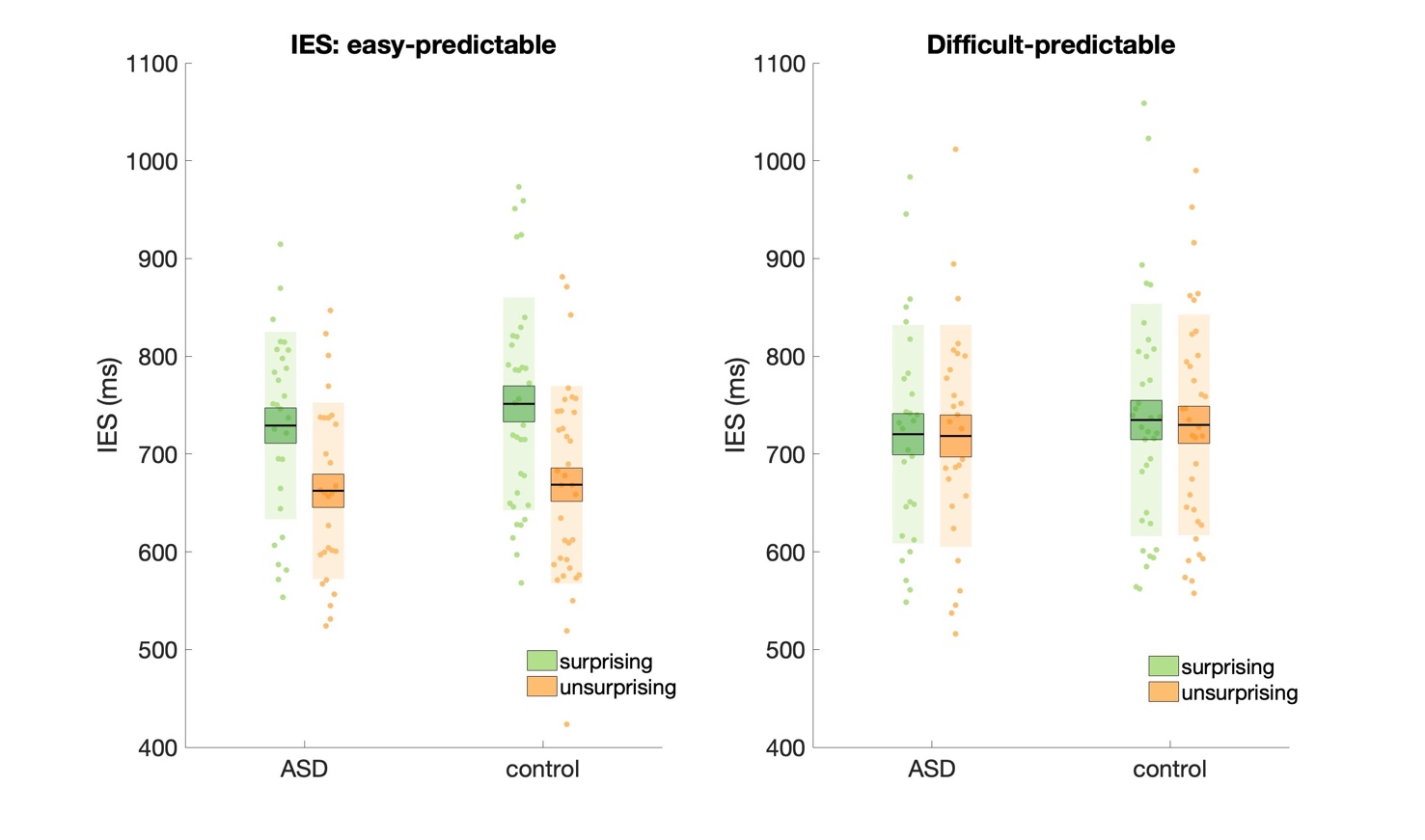


**Fig. S2. IES scores.** IES scores were lower for unsurprising trials for the easy but not the difficult predictable conditions. No differences were observed between groups. Data points indicate individual participants. The mean is the thick black horizontal line, and 1 standard error of the mean (SE) is represented by the shaded box around the mean. Standard deviation (SD) is the shaded region.

# Tables

**Table S1. Clinical information for the autistic individuals**

|  | Total sample (%)  N=28 |
| --- | --- |
| Major depression | 2 (7%) |
| Generalized anxiety disorder | 3 (11%) |
| Bipolar disorder | 1 (4%) |
| ADHD | 3 (11%) |
| Epilepsy | 1 (3%) |
| No current disorder* | 22 (78%) |

# References

1. Strange, B. A., Duggins, A., Penny, W., Dolan, R. J. & Friston, K. J. Information theory, novelty and hippocampal responses: Unpredicted or unpredictable? *Neural Networks* **18**, 225–230 (2005).

2. Harrison, L. M., Duggins, A. & Friston, K. J. Encoding uncertainty in the hippocampus. *Neural Networks* **19**, 535–546 (2006).

3. Townsend, J. T. & Ashby, F. G. Methods of modeling capacity in simple processing systems. in *Cognitive Theory Vol. III* 199–239 (Erlbaum Associates, 1978). doi:10.1163/_q3_SIM_00374.

4. Dienes, Z. Using Bayes to get the most out of non-significant results. *Front. Psychol.* **5**, 1–17 (2014).

5. Rouder, J. N., Morey, R. D., Speckman, P. L. & Province, J. M. Default Bayes factors for ANOVA designs. *J. Math. Psychol.* **56**, 356–374 (2012).

6. Rouder, J. N., Speckman, P. L., Sun, D., Morey, R. D. & Iverson, G. Bayesian t tests for accepting and rejecting the null hypothesis. *Psychon. Bull. Rev.* **16**, 225–237 (2009).

7. van Doorn, J. *et al.* The JASP Guidelines for Conducting and Reporting a Bayesian Analysis. *PsyArxiv Prepr.* 0–31 (2019) doi:10.31234/osf.io/yqxfr.

8. Wagenmakers, E. J. A practical solution to the pervasive problems of p values. *Psychon. Bull. Rev.* **14**, 779–804 (2007).

9. Van Den Bergh, D. *et al.* A tutorial on conducting and interpreting a bayesian ANOVA in JASP. *Annee Psychol.* **120**, 73–96 (2020).

10. Lee, M. D. & Wagenmakers, E. J. *Bayesian cognitive modeling: A practical course*. *Bayesian Cognitive Modeling: A Practical Course* (Cambridge University Press, 2013). doi:10.1017/CBO9781139087759.
